# Brassinosteroids Regulate OFP1, a DLT Interacting Protein, to Modulate Plant Architecture and Grain Morphology in Rice

**DOI:** 10.3389/fpls.2017.01698

**Published:** 2017-09-27

**Authors:** Yunhua Xiao, Dapu Liu, Guoxia Zhang, Hongning Tong, Chengcai Chu

**Affiliations:** ^1^State Key Laboratory of Plant Genomics and Center for Plant Gene Research, Institute of Genetics and Developmental Biology, Chinese Academy of Sciences, Beijing, China; ^2^University of Chinese Academy of Sciences, Beijing, China; ^3^National Key Facility for Crop Gene Resources and Genetic Improvement, Institute of Crop Sciences, Chinese Academy of Agricultural Sciences, Beijing, China

**Keywords:** DLT, grain shape, GSK2, leaf angle, OFP, plant height, rice

## Abstract

Brassinosteroids (BRs) regulate important agronomic traits in rice, including plant height, leaf angle, and grain size. However, the underlying mechanisms remain not fully understood. We previously showed that GSK2, the central negative regulator of BR signaling, targets DLT, the GRAS family protein, to regulate BR responses. Here, we identified Ovate Family Protein 1 (OFP1) as a DLT interacting protein. *OFP1* was ubiquitously expressed and the protein was localized in both cytoplasm and nucleus. Overexpression of *OFP1* led to enlarged leaf angles, reduced plant height, and altered grain shape, largely resembled *DLT* overexpression plants. Genetic analysis showed that the regulation of plant architecture by OFP1 depends on DLT function. In addition, we found *OFP1* was greatly induced by BR treatment, and OsBZR1, the critical transcription factor of BR signaling, was physically associated with the *OFP1* promoter. Moreover, we showed that gibberellin synthesis was greatly repressed in *OFP1* overexpression plants, suggesting OFP1 participates in the inhibition of plant growth by high BR or elevated BR signaling. Furthermore, we revealed that OFP1 directly interacts with GSK2 kinase, and inhibition of the kinase activity significantly promotes OFP1 protein accumulation in plant. Taken together, we identified OFP1 as an additional regulator of BR responses and revealed how BRs promote OFP1 at both transcription and protein levels to modulate plant architecture and grain morphology in rice.

## Introduction

Brassinosteroids (BRs) are a class of phytohormones playing important roles in regulating various aspects of plant growth and development. In the past decades, rapid progresses have been made regarding the hormone signaling in the model plant Arabidopsis. A nearly complete primary signaling pathway has been established from the receptor to the transcriptional factors involving multiple components including BRI1 receptor kinase, BAK1 co-receptor kinase, BKI1 receptor inhibitor kinase, BSK1 and CDG1 kinases, BSU1 phosphatase, BIN2 kinase, PP2A phosphatase, BZR1/BES1 transcriptional factors, and so on (Gruszka, 2013; Vriet et al., 2013). Among them, BIN2 is the central negative regulator which directly inhibits BZR1/BES1 activity (He et al., 2002; Yin et al., 2002). Recently, KIB1 was identified as a negative regulator of BR signaling responsible for BIN2 degradation, and multiple components including SINAT and DSK were identified as positive regulator of BR signaling participated in BZR1/BES1 degradation (Nolan et al., 2017; Yang and Wang, 2017; Yang et al., 2017; Zhu et al., 2017). In addition, a number of additional BIN2 targets as well as BES1 interacting proteins were reported, which are involved in diverse biological processes, further complemented the signaling network and greatly advanced our understanding of BR functional mechanisms (Hao et al., 2013; Youn and Kim, 2015).

In crop plant rice, BRs play critical roles in regulating plant height, leaf angle, and grain size. Because these characteristics are important agronomic traits that mostly considered in breeding activity, BRs are thought to have great potentials in agricultural improvement (Divi and Krishna, 2009). Several studies have tried to utilize BR-related plants or genes for crop engineering and obtained promising results. For example, a weak BR-deficient plant *osdwarf4*, or an *OsBRI1*-cossuppression plant could have greatly enhanced population yield when planting at high density due to their favorable erect leaves (Morinaka et al., 2006; Sakamoto et al., 2006). However, since rice has a distinct plant architecture from Arabidopsis, it remains largely unclear how BRs specifically regulate these traits in detail in rice. Consistently, although some counterparts of the BR primary signaling components including OsBRI1, GSK2 (OsBIN2), and OsBZR1 were identified and their function were verified or partially verified, several rice specific BR components were also reported, including DLT, LIC, SMOS1/RLA1, and OFP8 (Wang et al., 2008; Tong et al., 2009; Tong and Chu, 2012; Zhang et al., 2012; Yang et al., 2016; Hirano et al., 2017; Qiao et al., 2017).

OFPs, for short of Ovate Family Proteins, are plant specific transcription factors containing a common domain named OVATE or DUF623 (domain of unknown function) (Wang et al., 2007, 2011). In rice, it should be noted that there were two genome-wide characterization studies of OFPs, which adopted different numbering systems of the family members (Liu et al., 2014; Yu et al., 2015). Founding member of the family, *OVATE*, was cloned in tomato, which plays crucial roles in regulating fruit shape (Liu et al., 2002). A naturally existed nonsense mutation in the gene in tomato varieties leads to the shift of round-shape fruit to pear-like shape. Surprisingly, consequent studies in Arabidopsis revealed that loss-of-function plants of many single or multiple OFP members are phenotype-silent (Wang et al., 2007, 2011; Schmitz et al., 2015). In contrast, overexpression of some OFP members produced evident morphology changes, suggesting the high redundancy among the family members. Overexpression of *AtOFP1* in Arabidopsis produced dwarf statue due to the repression of gibberellin (GA) synthesis (Wang et al., 2007). AtOFP1 directly targets *GA20ox-1*, a GA biosynthetic gene, to inhibit its expression. Similarly, overexpression of *OsOFP2* in rice also inhibited GA synthesis and decreased plant height likely by regulating *GA20ox-7* (Schmitz et al., 2015). In addition, OFPs tend to generally interact with BELL- and KNOX-like proteins to regulate plant growth and development (Hackbusch et al., 2005; Schmitz et al., 2015). Recently, OFP8, corresponding to OFP30 in Liu et al. (2014), was shown to be involved in BR responses (Yang et al., 2016). Enhanced expression of the gene caused by T-DNA insertion containing 35S enhancer produced obviously enlarged leaf angles and increased BR responses. The protein localization was modulated by GSK2 phosphorylation, similar as the regulation of BZR1/BES1 by BIN2 (Yang et al., 2016).

More and more studies suggested that functions of BRs could be variable depending on species, tissues, hormone levels, developmental stages, and cell types (Singh and Savaldi-Goldstein, 2015; Tong and Chu, 2016; Unterholzner et al., 2016). For example, BRs were reported to repress stomata development in cotyledons but promote the process in hypocotyls in Arabidopsis (Gudesblat et al., 2012; Kim et al., 2012). As growth-promoting hormones, BRs promote GA accumulation by inducing the expression of GA biosynthetic genes to stimulate cell elongation under physiological conditions (Tong et al., 2014; Unterholzner et al., 2015), however, sufficient high BR level or signaling will inhibit cell elongation and plant growth partially by repressing GA synthesis in rice (Tong et al., 2014). The mechanisms underlying this kind of functional specificities of BRs remain largely unclear.

In this study, we identified OFP1 as a DLT interacting protein by yeast two-hybrid screening, and found that OFP1 can also interact with GSK2 kinase. We showed that OFP1 was regulated by BRs at both transcription and protein levels, and the regulation was closely linked to the core BR signaling components involving GSK2, OsBZR1, and DLT. Our results revealed the positive roles of OFP1 in regulating BR responses to modulate plant architecture and grain morphology.

## Materials and Methods

### Plant Materials, Growth Conditions, and Chemical Treatment

*Japonica* cultivars Zhonghua 11 (ZH11) or Dongjin (DJ) was used as the wild type (WT) for the transgenic analyses. Plants were grown on soil in field or in greenhouse or on 0.5x Murashige and Skoog (1/2MS) medium in a conditioned growth chamber at 30°C for 10 h (day) and 24°C for 14 h (night). For BR induction experiments, young seedlings were transferred to 1/2MS medium supplemented with various concentrations of brassinolide (BL, one of the active BRs) for 48 h. For lamina inclination assay, ethanol (1 μL) containing various amount of BL was spotted onto the top of lamina after 2-day germination and 3-day growth at 30°C (Tong and Chu, 2017). Images were taken after 3-day incubation, and the angles of lamina joint bending were measured using IMAGEJ software^1^. For bikinin treatment, 1-week-old seedlings were grown in 1/2MS supplemented with or without 30 μM chemical for 3 days before sampling.

### Vector Constructions and Transgenic Analysis

The DNA sequence containing the promoter and coding sequence of *OFP1* was cloned into *pCAMBIA2300* vector for *OFP1* overexpression (*OFP1-OE* or *1o*). A *pCAMBIA2300-35S-GFP* vector was used for *OFP1-GFP* construction. A *pCAMBIA1300-35S-Flag* vector was used for *OFP1-Flag* overexpression (*OFP1-Flag-OE* or *1Fo*). *OFP1* promoter (2-kb) was introduced into *pCAMBIA2391Z* vector for *OFP1p:GUS* construction. CRISPR/Cas9 knock-out vectors were constructed following previous reports (Ma and Liu, 2016). Primer sequences used for vector constructions and additional plasmid information are listed in the Supplementary Table 1. Sequences were introduced into vectors by either in-fusion cloning strategy (Clontech) or traditional cut-ligation cloning method. These constructs were used to transform rice plant or to infiltrate tobacco leaf epidermis cells by *Agrobacterium*-mediated method (Sparkes et al., 2006).

### Protein: Protein Interaction Analyses

Yeast two-hybrid tests were performed following standard procedures descried by manufacturer (Clontech). BiFC (bimolecular fluorescence complementation) analyses were performed following the method described previously (Waadt et al., 2008). Rice protoplast preparation and plasmid transfection were performed according to the previous method (Bart et al., 2006). Fluorescence was observed on a confocal fluorescence microscope (Leica TCS SP6). Split-luciferase complementation assays were performed in tobacco leaves as described (Chen et al., 2008). Chemiluminiscence was photographed using a imaging system equipped with a cold CCD (NightOWL II LB983 with indigo software). See Supplementary Table 1 for the information of the primer sequences and plasmids used for the vector constructions prepared for the above analyses.

### Gene Expression and Promoter Activity Analysis

RNA was isolated using Trizol reagent (Invitrogen) according to manufacturer’s instruction. The first-strand cDNA was synthesized using a Revertase Transcription kit (Toyoba). Quantitative RT-PCR (qRT-PCR) was performed on a real-time PCR detection system following manufacturer’s instructions (Bio-Rad CFX96). Rice *Ubiquitin2* gene (*UBQ*) was used as internal control for all analyses. Primer sequences are listed in the Supplementary Table 2. GUS staining was performed according to previous description (Tong et al., 2009).

### Immunoblotting and ChIP-qPCR Analysis

Commercialized anti-Flag (Sigma) and anti-ACTIN (Abmart) antibodies were used for immunoblotting analyses. Commercialized anti-OsBZR1 antibody (BPI) was used for ChIP (chromatin immunoprecipitation) analysis. ChIP-qPCR (ChIP followed by quantitative PCR analysis) was performed according to previous description (Tong et al., 2014). Primer sequences are listed in the Supplementary Table 2.

### GA Measurement

About 4 g of shoots was harvested from the rice seedlings for GA measurements. Quantification of endogenous GAs was performed as described (Chen et al., 2012).

## Results

### OFP1 Interacts with DLT in Yeast

**FIGURE 1:**
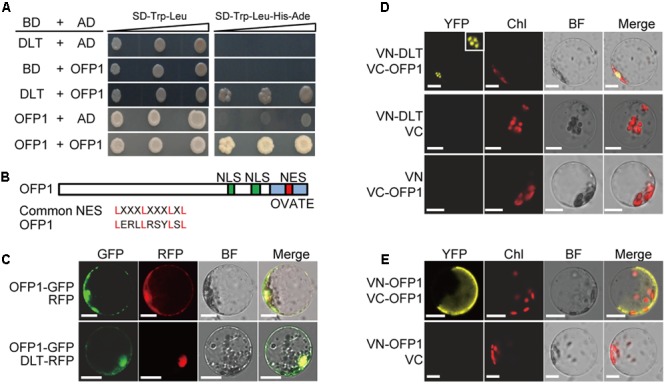
OFP1 interacts with DLT and itself in yeast and plant cells. **(A)** Yeast two-hybrid analyses of the indicated proteins on minimal synthetic defined media containing -Leu/-Trp or -Leu/-Trp/-His/-Ade dropout supplements. **(B)** Schematic diagram of OFP1 protein with NES, NLS, and OVATE domain indicated. Alignment of the putative NES with the common NES sequence was shown. **(C)** Subcellular co-localization of OFP1-GFP with RFP or DLT-RFP. BF, bright field. Scale bars = 10 μm. **(D,E)** BiFC analysis of the interactions between the indicated proteins. Nucleus was magnified to show the punctate structure. Chl, chloroplast fluorescence; BF, bright field. Scale bars = 10 μm.

We previously showed that GSK2 kinase, the central negative regulator of BR signaling, targets DLT, the GRAS family transcription factor, to regulate downstream BR responses (Tong et al., 2012). To further explore the functional mechanism of the proteins, we performed extensive yeast two-hybrid screenings of the interacting proteins using DLT as bait. By this approach, we identified OFP1 (Ovate Family Protein 1) as one of DLT-interacting proteins from the yeast library prepared using mixed rice tissues. Full length OFP1 prey vector (OFP1-AD) was further constructed and the interaction was confirmed in yeast (**Figure 1A**). In addition, we found that when OFP1 was used as both bait and prey, the interaction also occurred (**Figure 1A**), suggesting that OFP1 may form homo-dimer or oligomer to function.

### Subcellular Localization of OFP1 and Verification of the Interaction in Plant Cells

OFP1 contains both the putative NLS (nuclear localization signal) and NES (nuclear export signal) (**Figure 1B**). To analyze the protein subcellular localization, we fused the protein with GFP and introduced the corresponding vector into rice protoplasts. Consistently, microscopic observation revealed that OFP1-GFP fusion proteins were localized in both cytoplasm and nucleus, and can be co-localized with DLT-RFP distributed in nucleus (**Figure 1C**), supporting the possibility that they may interact in plant cells.

We then further verified their interactions by BiFC analysis in rice protoplasts. Different combinations of the proteins fused with either C-terminal or N-terminal of the split Venus YFP (VC or VN) were introduced into the rice protoplast cells, and the reconstituted fluorescence signals were detected using a laser confocal microscope. While as negative controls no obvious signal was detected between any of the fusion proteins and the corresponding empty split YFP partial protein, we observed the strong interaction between VC-OFP1 and VN-DLT in nucleus (**Figure 1D**). Interestingly, the interacting signals of both protein pairs were mostly spotted in punctate-structures, reminiscent of nuclear bodies which could be formed due to the assembling and spatial-specific distribution of protein complex (**Figure 1D**). This distinct pattern also indicated that the fluorescence was not false-positive signal and the interaction was indeed occurred. In addition, we also detected the clear interaction between OFP1 itself (**Figure 1E**). Notably, in most observed cells, the interacting signals were diffusely distributed in part of cells, which appeared to be mainly in cytoplasm, with unclear reason (**Figure 1E**).

### Molecular Characterization of OFP1

**FIGURE 2:**
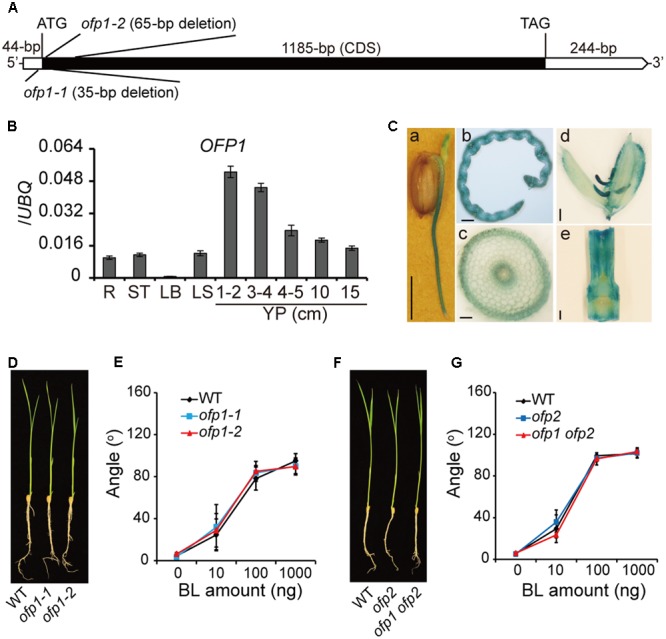
Characterization of *OFP1* expression and the *ofp1* knockout mutants. **(A)** Schematic diagram of *OFP1* gene structure. UTR and coding sequence were indicated. Mutation sites of *ofp1* alleles produced by CRISPR/CAS9 technology were shown. **(B)** Relative expression levels of *OFP1* in various rice tissues tested by qRT-PCR. R, root; ST, stem; LB, leaf blade; LS, leaf sheath; YP, young panicle. Numbers indicate the panicle length (cm). *UBQ* gene was used as internal reference. *n* = 3, bar = SD. **(C)** *OFP1* promoter activity analysis in various tissues evaluated by GUS reporter. (a) coleoptile and root on germinated seed; (b) transverse section of young leaf blade; (c) transverse section of root; (d) spikelet; (e) internode. Scale bars = 5 mm in (a), 100 μm in (b, c), 1 mm in (d, e). **(D)** Seedling morphology of *ofp1* knockout mutants. **(E)** Lamina bending analysis of the plants in **(D)** in response to various amount of BL. *n* = 10, bar = SD. **(F)** Seedling morphology of *ofp2* and *ofp1 ofp2* knockout mutants. **(G)** Lamina bending analysis of the plants in **(F)** in response to various amount of BL. *n* = 10, bar = SD.

According to rice genome annotation project^2^, *OFP1* (*LOC_Os01g12690*) is intronless and contains 1185-bp coding sequence, 44-bp 5′-UTR and 244-bp 3′-UTR (**Figure 2A**). We analyzed the gene expression pattern by qRT-PCR, and found *OFP1* was ubiquitously expressed in various rice tissues, preferentially higher in young panicles (**Figure 2B**). We also evaluated the gene promoter activity using GUS reporter and successfully detected the signals in various tissues of *OFP1p:GUS* transgenic plants, but mainly at young stages of the tissues (**Figure 2C**).

### Knock-out *ofp1* Mutants and *ofp1 ofp2* Double Mutants Showed No Obvious Morphological Phenotype

We produced the loss-of-function mutants of *OFP1* by CRISPR/Cas9 gene-editing technology. A segment located at the 5′-end of the gene coding sequence was targeted for the editing, and a number of independent homozygous mutant lines were obtained. Two distinct alleles, *ofp1-1* and *ofp1-2*, were selected for the further analysis. These alleles contained 35- and 65-bp deletions around the initial codon respectively, leading to the frameshift afterward of the sequence, suggesting that both are knockout mutants (**Figure 2A**). However, we failed to observe the morphological difference between *ofp1* mutants and WT at both seedling stage and mature stage (**Figure 2D**). Both the mutants also exhibited indistinguishable BR sensitivity compared with the WT in lamina bending assay (**Figure 2E**). We further generated *ofp1 ofp2* double mutant by similar strategy, yet the homozygous mutant plants still showed no obviously phenotypes under normal growth conditions as well as unaltered BR sensitivity in lamina bending assay (**Figures 2F,G**).

### *OFP1* Transgenic Plants Exhibit Significantly Altered Plant Architecture and Grain Morphology

**FIGURE 3:**
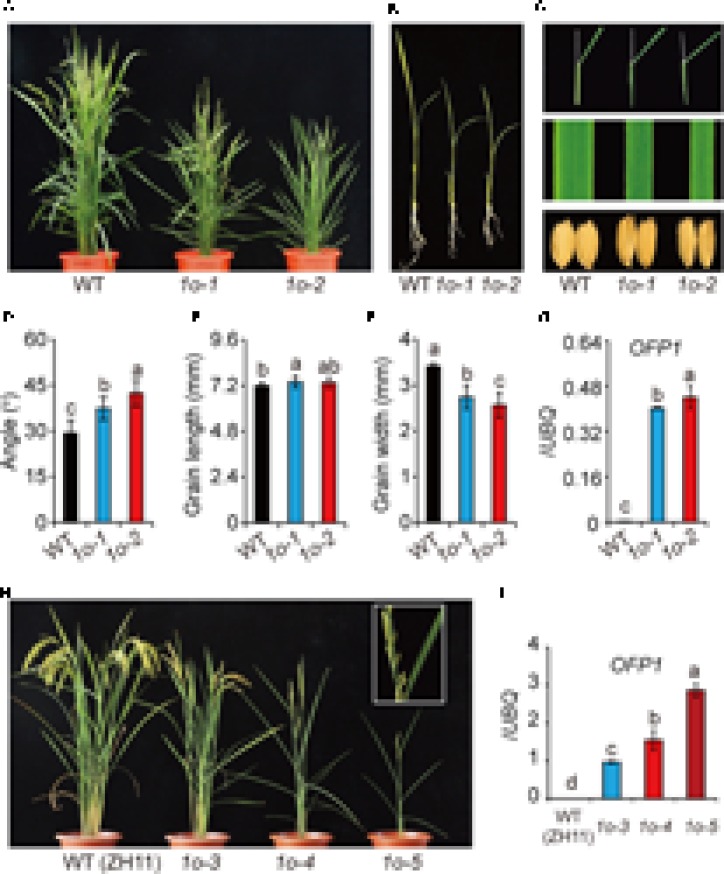
Phenotype analysis of *OFP1* overexpression plants. **(A)** Gross morphology of two representing *OFP1* overexpression plants in DJ background, designated as *1o-1* and *1o-2* for short, at the reproductive stage. **(B)** Seedling morphology of *1o-1* and *1o-2* compared with WT. **(C)** Detailed comparison of leaf angle, leaf width, and grain morphology. Statistical data of the leaf angle **(D)**, grain length **(E)**, and grain width **(F)** of the plants. *n* = 12 in **(D)**, *n* = 20 in **(E,F)**, bar = SD. Different letters above the columns indicate statistically significant differences between groups (Fisher’s LSD, *p* < 0.05). **(G)** Relative *OFP1* expression in the plants tested by qRT-PCR. *UBQ* gene was used as internal reference. *n* = 3, bar = SD. Different letters above the columns indicate statistically significant differences between groups (Fisher’s LSD, *p* < 0.05). **(H)** Phenotypes of three representing *OFP1* overexpression plants in ZH11 background, designated as *1o-3*, *1o-4*, and *1o-5* for short. Enlarged picture showed the development of sterile seeds and roll leaves in the severe line. **(I)** Relative *OFP1* expression in the plants tested by qRT-PCR. *UBQ* gene was used as internal reference. *n* = 3, bar = SD. Different letters above the columns indicate statistically significant differences between groups (Fisher’s LSD, *p* < 0.05).

There are 33 OFPs in rice, and different OFP members could function with high redundancy, as strongly suggested in Arabidopsis (Wang et al., 2011; Liu et al., 2014). In this case, transgenic analysis was generally used to analyze the protein function. To this end, the whole *OFP1* gene containing 2-kb upstream promoter and coding region was introduced into either Dongjing (DJ) or Zhonghua 11 (ZH11), two different *Japonica* WTs. Strikingly, although with different extents due to the various gene expression levels, most of the transgenic plants (*OFP1-OE*, designated as *1o* for short in Figures) showed obvious phenotypes in both WT backgrounds (**Figure 3**). Unless specified, we majorly used transgenic lines in DJ background for our analysis, since we obtained the stable homozygous lines at the first place.

*OFP1-OE* had narrow leaves, enlarged leaf angles, decreased plant height, reduced tiller number, and long and narrow seeds (**Figures 3A–F,H**). The grain morphology was remarkably changed due to the greatly increase of the length/width ratio (**Figures 3C,E,F**). The severe lines are sterile, and have roll leaves and significantly decreased tiller numbers (**Figure 3H**). Most of these phenotypes, including the typical BR-enhanced phenotypes, largely resembled *DLT*-overexpression plants (*Do*), featured with the significantly narrowed leaves and seeds which could be, at least partially, BR-independent (Tong et al., 2012). Severities of the phenotypes were obviously consistent with the gene expression levels in the transgenic plants (**Figures 3A–I**), suggesting the involvement of *OFP1* in controlling these phenotypes. However, we surprisingly found that the gene was remarkably over-expressed in the positive transgenic plants (**Figures 3G,I**). While the reason is unclear, one possibility is that there exists additional suppressor elements outside the selected promoter region.

### OFP1 Enhanced BR Responses and Required DLT to Regulate Plant Architecture

**FIGURE 4:**
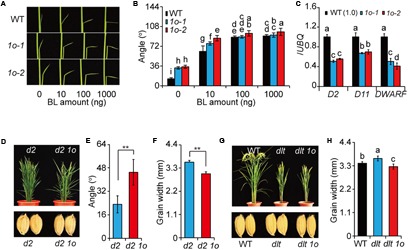
*OFP1* overexpression plants have enhanced BR responses. **(A,B)** Lamina bending analysis of the BR sensitivity of *1o-1* and *1o-2*. Statistical data were shown in **(B)**. *n* = 10, bar = SD. Different letters above the columns indicate statistically significant differences between groups (Fisher’s LSD, *p* < 0.05). **(C)** Relative expression of the BR synthetic genes in *1o-1* and *1o-2*. Expression in WT was set as 1.0. *UBQ* gene was used as internal reference. *n* = 3, bar = SD. Different letters above the columns indicate statistically significant differences between groups (Fisher’s LSD, *p* < 0.05). *OFP1* overexpression in *d2* background **(D)** and statistical data of the leaf angle **(E)** and grain width of the plants **(F)**. *n* = 5 in **(E)**, *n* = 20 in **(F)**, bar = SD, ^∗∗^*p* < 0.01 in Student’s *t*-test. *OFP1* overexpression in *dlt* background **(G)** and statistical data of the grain width of the plants **(H)**. *n* = 20, bar = SD. Different letters above the columns indicate statistically significant differences between groups (Fisher’s LSD, *p* < 0.05).

We further tested the BR sensitivity of the plants by lamina inclination assay. *OFP1-OE* showed increased leaf angles than WT either with or without BR (**Figures 4A,B**). To exclude the possibility that the enhanced leaf angles were caused by increased BR levels, we analyzed the expression of BR synthetic genes including *D2*, *D11*, and *DWARF* and found that all of them have markedly decreased expression in *OFP1-OE* (**Figure 4C**), similar to that in *Do* plants (Tong et al., 2012). This result also implied that the BR synthesis was inhibited by enhanced BR responses in a feedback manner in *OFP1-OE*. Combined with the phenotypes, these results demonstrated that *OFP1-OE* plants have activated BR responses.

To further analyze the OFP1 functions, we introduced *OFP1* into *d2*, a BR synthesis mutant (Hong et al., 2003), by transgenic method, and found that *d2 OFP1-OE* showed obviously enlarged leaf angles and decreased grain width compared with *d2* (**Figures 4D–F**). We also introduced *OFP1* into *dlt* mutant, and found *dlt OFP1-OE* showed similar morphology as *dlt*, suggesting that OFP1 required DLT to regulate plant architecture (**Figure 4G**). It should be noted that *OFP1-OE* can obviously suppress the grain width of *dlt* mutant (**Figures 4G,H**), demonstrating that the protein is functional in the transgenic plant. Considering BR has relatively minor roles in regulating grain width (Tanabe et al., 2005), this result indicated that OFP1 and DLT have differential functional relationship in different tissues or biological processes. Taken together, these results strongly suggested that OFP1 positively regulates BR responses.

### BR Promotes *OFP1* Expression and OsBZR1 Binds to *OFP1* Promoter

**FIGURE 5:**
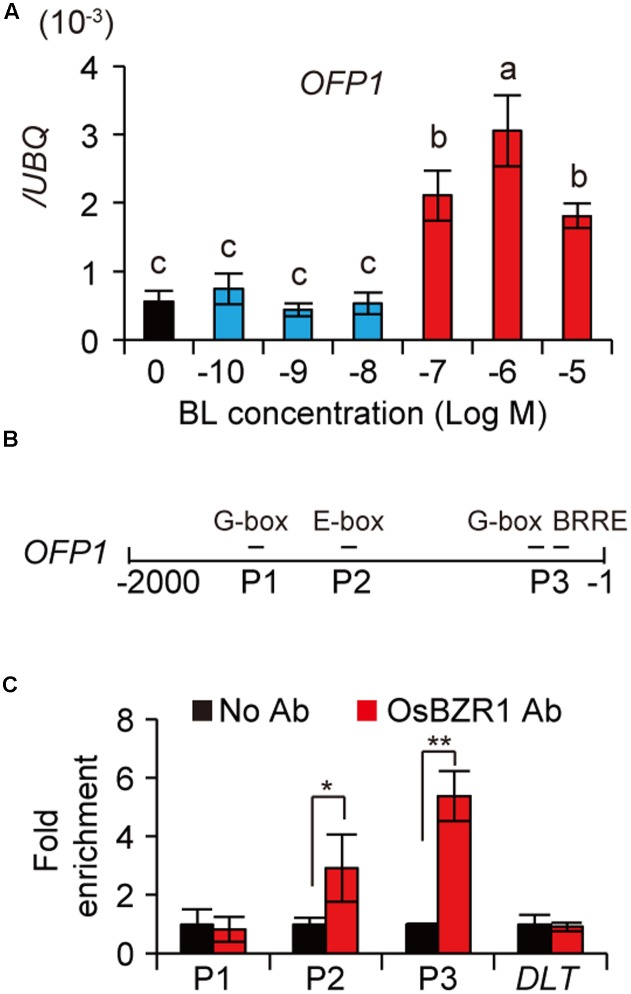
BR induces OFP1 transcription by OsBZR1 binding. **(A)** Expression analysis of *OFP1* under the treatment of BL with different concentrations. *UBQ* gene was used as internal reference. *n* = 4, bar = SD. Different letters above the columns indicate statistically significant differences between groups (Fisher’s LSD, *p* < 0.05). **(B)** Schematic show of *OFP1* promoter. Putative BZR1 targeted *cis*-elements and the segments (P1–P3) for ChIP-qPCR analysis were indicated. **(C)** ChIP-qPCR analysis of OsBZR1 binding on *OFP1* promoter using OsBZR1 antibody. A segment reside in DLT coding region was used as control. *n* = 3, bar = SD, ^∗^*p* < 0.05, ^∗∗^*p* < 0.01 in Student’s *t*-test.

We have performed RNA-seq analysis to compare the differentially expressed genes between high BL and low BL treated rice plants. In our available data, we found *OFP1* was differentially over-expressed in 10^-6^ M BL treated rice seedlings, but not in 10^-9^ M BL treated materials. To confirm this result, we treated WT plants using various concentrations of BL, and then analyzed the expression of *OFP1* by qRT-PCR. Consistently, the results showed that *OFP1* was markedly induced by application of BR above 10^-7^ M, but not induced under lower BR concentrations (**Figure 5A**). In addition, our available ChIP-seq data using OsBZR1 antibody enriched OsBZR1 protein on the promoter region of *OFP1* with 3.28-fold increase in WT rice seedlings. Accordingly, we found *OFP1* promoter contains multiple *cis*-elements that have been suggested to be bound by BZR1 (**Figure 5B**) (Sun et al., 2010; Yu et al., 2011). The association between OsBZR1 and the *OFP1* promoter was further confirmed by ChIP-qPCR (**Figure 5C**). Compared with the samples with no antibody supplemented, OsBZR1 antibody can significantly pull down the DNA segments containing the putative BZR1 binding elements (**Figure 5C**).

### OFP1 Is Involved in BR Inhibition of GA Synthesis and Plant Growth

**FIGURE 6:**
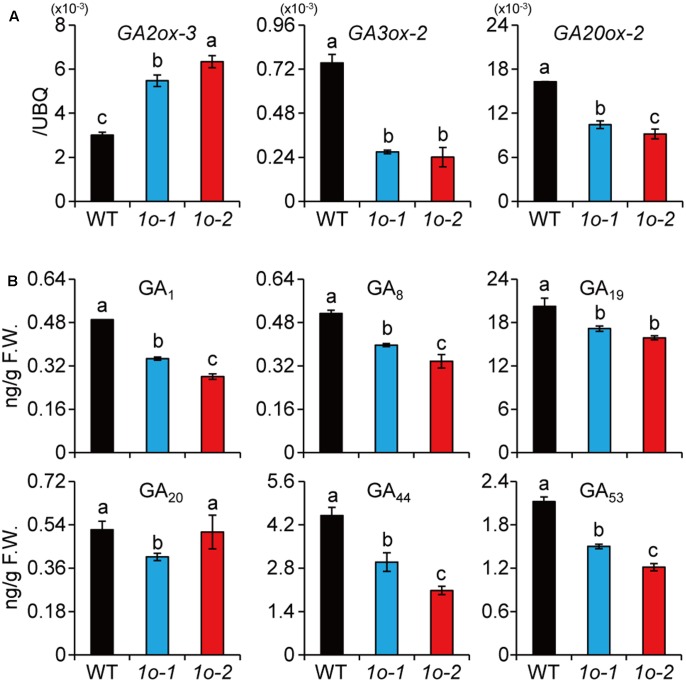
*OFP1* overexpression plants have decreased GA synthesis. **(A)** Relative expression of the GA metabolism genes in *1o-1* and *1o-2*. Expression in WT was set as 1.0. *UBQ* gene was used as internal reference. *n* = 3, bar = SD. Different letters above the columns indicate statistically significant differences between groups (Fisher’s LSD, *p* < 0.05). **(B)** Quantification of GA levels in *1o-1* and *1o-2*. F.W., fresh weight. *n* = 3, bar = SD. Different letters above the columns indicate statistically significant differences between groups (Fisher’s LSD, *p* < 0.05).

In Arabidopsis, it was shown that AtOFP1 directly suppresses GA synthetic gene to repress plant growth (Wang et al., 2007). Our previous study also suggested that high BR levels or enhanced BR signaling will repress GA level to inhibit plant growth in rice (Tong et al., 2014). The specific induction of *OFP1* by high BR (**Figure 5A**) and the strongly repressed growth of *OFP1-OE* plants (**Figures 3A,B**) prompted us to test whether OFP1 was involved in this process. Indeed, we found *GA3ox-2* and *GA20ox-2*, two GA synthetic genes, had decreased expression, whereas *GA2ox-3*, the GA inactivation gene, had increased expression in *OFP1-OE* (**Figure 6A**). Accordingly, GA_1_, the major bioactive GA form in young seedlings, was significantly decreased in the plants (**Figure 6B**), consistent with their decreased seedling height (**Figure 3B**). Actually, all the GA forms quantified were decreased in *OFP1-OE* plants (**Figure 6B**), reminiscent of the hormonal profiles obtained from high-BL-treated plants as shown previously (Tong et al., 2014). These results indicated that OFP1 is involved in the BL-induced GA repression and growth inhibition, depending on BR concentrations or plant tissues.

### OFP1 Protein Stability Was Regulated by BR and Development Stages

**FIGURE 7:**
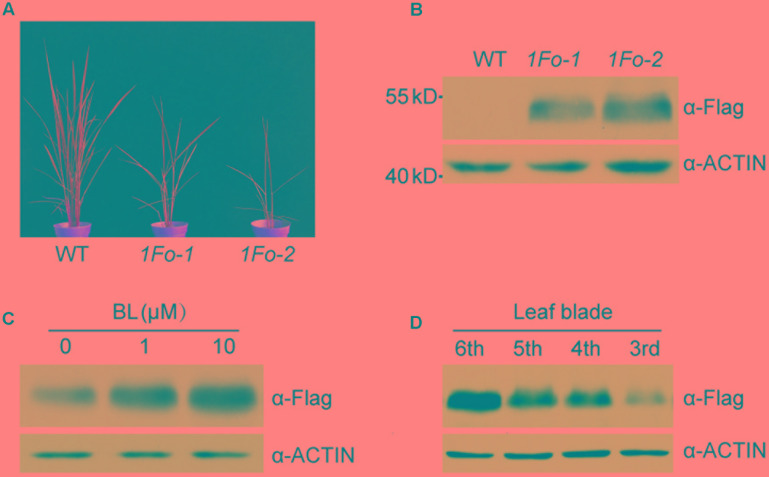
OFP1 protein was regulated by BR and developmental stage. **(A)** Gross morphology of two representative *OFP1-Flag* overexpression plants, designated as *1Fo-1* and *1Fo-2* for short. **(B)** Immunoblotting analysis of OFP1-Flag fusion proteins in the indicated rice plants. Rice ACTIN protein was blotted as loading reference. **(C)** Effects of BL on the protein stability of OFP1-Flag in *1Fo-2*. Rice ACTIN protein was blotted as loading reference. **(D)** Immunoblotting analysis of OFP1-Flag fusion proteins in different leaf blades in *1Fo-2.* Rice ACTIN protein was blotted as loading reference.

To test whether OFP1 was regulated at protein level, we fused the coding sequence with Flag tag and introduced into the WT under 35S promoter (*OFP1-Flag-OE*, designated as *1Fo* for short in Figures). We obtained a collection of the overexpression plants which showed similar phenotypes as above described *OFP1-OE* plants, including enlarged leaf angle, reduced plant height, and altered grain shape (**Figure 7A**). Immunoblotting analysis using Flag antibody can detect the protein expression in the corresponding plants, demonstrating the fusion proteins are functional (**Figure 7B**). We treated the plants with different concentrations of BL, and found the protein was gradually accumulated (**Figure 7C**). We also tested the OFP1 protein in different leaves representing the different developmental stages, and revealed that the protein was highly accumulated in young leaves, but gradually decreased in old leaves (**Figure 7D**). These results suggested that OFP1 was dynamically regulated by hormone levels and development stages.

### GSK2 Interacts with OFP1 and Regulates OFP1 Stability

**FIGURE 8:**
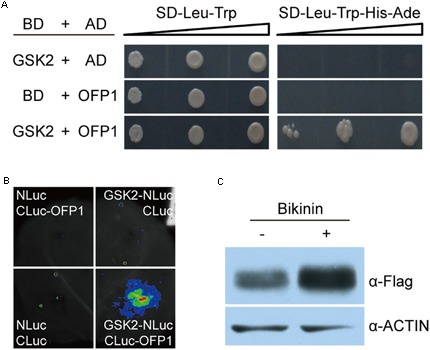
GSK2 interacts with OFP1 and regulates OFP1 stability. **(A)** Yeast two-hybrid analysis of the interaction between GSK2 and OFP1. **(B)** Split luciferase complementation analysis of the interaction between GSK2 and OFP1. **(C)** Effects of bikinin, the GSK3-like kinase inhibitor, on the protein stability of OFP1-Flag in *1Fo-2*. Rice ACTIN protein was blotted as loading reference.

GSK3-like kinases, as the central negative regulators of BR signaling, can interact with many transcriptional factors to modulate their stability and activity (Youn and Kim, 2015). The promotion of OFP1 stability by BR prompted us to test whether GSK2 can directly interact with OFP1 to regulate this process. Indeed, yeast two-hybrid analysis revealed the obviously interaction between GSK2 bait and OFP1 prey (**Figure 8A**). Surprisingly, we can rarely or only weakly observe the interaction between GSK2 and OFP1 by BiFC analysis in rice protoplasts. One possibility is that the interaction is unstable in rice, considering GSK2 might promote OFP1 degradation. Alternatively, we adopted split-luciferase complementation assay to analyze their interaction in tobacco leaves (**Figure 8B**). When the GSK2 fused with N-terminal of luciferase protein (GSK2-NLuc) was co-expressed with the OFP1 fused with C-terminal of luciferase protein (CLuc-OFP1), the interaction signals were evidently detected. No signal was detected when the GSK2-NLuc was co-expressed with the empty CLuc protein (**Figure 8B**). Thus, GSK2 indeed can interact with OFP1 in plant.

To explore whether GSK2 regulates OFP1 stability, we treated *OFP1-Flag-OE* plants using bikinin, a specific chemical inhibitor of GSK3-like kinase activity, and analyzed the OFP1 protein level. Compared with the plants without treatment, we found OFP1 was significantly accumulated (**Figure 8C**). Taken together, these results suggested that BR enhances OFP1 protein stability by inhibiting GSK2.

## Discussion

Our study identified OFP1 as an additional positive player of BR responses by interacting with GSK2, OsBZR1, and DLT. We proposed that (1) at transcription level, BR induces *OFP1* expression through OsBZR1, (2) at protein level, BR enhances OFP1 stability by inactivate GSK2, and (3) OFP1 activity partially relies on the physical interaction with DLT. In addition, our results suggested the novel roles of OsBZR1-induced *OFP1* that required for responding to elevated BR.

Functional characterization of OFPs progressed slowly, largely due to the high redundancy among the family members (Wang et al., 2011). To our knowledge, there is no phenotype-evident knockout plants of any OFP members reported in both rice and Arabidopsis. In Arabidopsis, T-DNA insertion *ofp* mutants including *ofp1*, *ofp4*, *ofp8*, *ofp10*, *ofp15*, and *ofp16*, all showed no visible phenotype under normal growth condition. In addition, the double mutants including *ofp1 ofp4* and *ofp15 ofp16* also showed no obvious phenotype (Wang et al., 2011). This seemed to be also the case in rice. The *ofp1* knockout mutant, as well as *ofp1 ofp2* double mutant, didn’t produce the expected phenotypes (**Figure 2**). However, knock-down of *OFP8/OFP30* by RNAi strategy indeed led to BR-related phenotypes (Yang et al., 2016). Although it’s unclear whether other members were non-specifically targeted, this result at least suggested the native function of OFPs in regulating BR responses. On the other hand, *OFP* genes tend to be flexibly regulated by various hormones (Yu et al., 2015), and overexpression of *OFPs* usually resulted in obvious morphological changes, suggesting their roles in regulating hormone responses. The extensively interaction between OFPs and KNOX proteins further implied this speculation, considering the crucial roles of KNOXs in regulating hormone balance to maintain meristem activity (Hackbusch et al., 2005; Tsuda et al., 2014). Given the distinct phenotypes of the *OFP1* overexpression plants, and the remarkable regulation of OFP1 transcription and protein by BRs, our study should, at least partially, revealed the function of the native protein in plant.

OFP1 function is closely related to DLT, both positively regulate BR responses. The overexpression plants of the two genes are highly similar. It should be mentioned that although *dlt* mutant showed decreased tiller number, the severe *DLT*-overexpression lines could also have decreased tiller number, similar as *OFP1-OE*. Genetic analysis showed that OFP1 function in regulating plant architecture, but not the grain width, is dependent on DLT (**Figure 4**). It should be mentioned that the significant regulation of grain width by DLT could be partially independent of BR response, since the typical BR mutants have little alteration of grain width (Tanabe et al., 2005). Accordingly, OFP1 also has marked effect on grain width, further implying the functional relevance between OFP1 and DLT. Notably, the regulation of grain shape (length/width ratio) by OFP1 and DLT in rice was somewhat reminiscent of *OVATE* or *OVATE*-like gene in regulating the fruit shape in both tomato and pepper (Liu et al., 2002; Tsaballa et al., 2011), potentially suggesting a general role of OFPs in regulating seed development. A bold speculation is that OFPs could have indispensable regulatory roles in plant growth and development, thus plant has developed highly redundant system to maintain the normal function of the family.

Although BRs were known to be a class of growth-promoting hormones, it was found that most BR-enhanced plants didn’t have increased plant height, and many of them actually exhibited decreased plant height in rice (Wang et al., 2008; Li et al., 2009; Tanaka et al., 2009; Tong et al., 2012). Consistently, exogenous high BR treatment also inhibits plant seedling growth. We previously discovered that this is because high BR or increased BR signaling turned to repress GA synthesis (Tong et al., 2014; Che et al., 2015). These observations suggested that BR balance is very important for plant growth. In this study, we revealed that OFP1 should play a role in this process. Overexpression of *AtOFP1* in Arabidopsis, or *OsOFP1* or *OsOFP2* or *OsOFP8* in rice, all leads to greatly reduced plant height (Wang et al., 2007; Schmitz et al., 2015; Yang et al., 2016). AtOFP1 directly targets *GA20ox-1* to suppress GA synthesis, and OsOFP2 was also suggested to regulate *GA20ox-7* to suppress GA synthesis. Measurement of GA contents in rice *OFP1* overexpression plants demonstrated the decreased GA levels of basically all GA forms (**Figure 6B**). Notably, this profile is very similar to that in the high BR treated plants, which also contained decreased levels of basically all quantified GA forms (Tong et al., 2014). Actually, the *OFP1* overexpression plants largely mimicked the high BR treated WT plants, including enlarged leaf angles and decreased plant height. Altogether, OFP1 should be involved in inhibition of GA levels and plant growth elicited by either high BR levels or elevated BR signaling.

## Conclusion

Elevated BR signaling will induce *OFP1* expression by OsBZR1, and also promote protein stability by inhibiting GSK2, leading to activation of OFP1, which interacts with DLT factors and targets downstream genes, including GA metabolism genes, to regulate plant architecture and grain morphology in rice.

## Author Contributions

YX and DL performed the study with the assistance of GZ and HT. HT, YX, and CC analyzed the data and wrote the manuscript. HT and CC conceived and supervised the study.

## Conflict of Interest Statement

The authors declare that the research was conducted in the absence of any commercial or financial relationships that could be construed as a potential conflict of interest.

## Footnotes

1 http://rsb.info.nih.gov/ij

2 http://rice.plantbiology.msu.edu
