# Supplementary material for: Brassinosteroids Regulate OFP1, a DLT Interacting Protein, to Modulate Plant Architecture and Grain Morphology in Rice
Source: Front Plant Sci. 2017 Sep 27;8:1698. doi: 10.3389/fpls.2017.01698 (PMC5623909; doi:10.3389/fpls.2017.01698)
Supplement: Supplementary file 1 [file Table_1.pdf]

Supplemental Table 1. Primers used for vector constructions.

**Name**	**Forward (5'-3')**	**Reverse (5'-3')**	**vector**
OFP1-AD	CATCGATACGGGATCCATATGGCGATGGATCATCGT	TCATCTGCAGCTCGAGCTAGCCGCCATGGAGGAC	*pGADT7*
DLT-AD	GAATTCATGTTGGCGGGTTGCTCGTTCTCGT	TCTAGATTAGCTTTGCTGAGAATGTGATGCTG	*pGADT7*
OFP1-BD	CATGGAGGCCGAATTCATGGCGATGGATCATCGT	GCAGGTCGACGGATCCCTAGCCGCCATGGAGGAC	*pGBKT7*
DLT-BD	GAATTCATGTTGGCGGGTTGCTCGTTCTCGT	CTGCAGTTAGCTTTGCTGAGAATGTGATGCTG	*pGBKT7*
GSK2-BD	GAATTCCAGCTTTGCCACATGGACCAGCCGGC	CTGCAGTTAGCTCCCAGTATTGAAG	*pGBKT7*
VN-OFP1	GCCTACTAGTGGATCCATGGCGATGGATCATCGT	GAGCGGTACCCTCGAGCTAGCCGCCATGGAGGAC	*pVYNE(R)*
VN-DLT	GCCTACTAGTGGATCCATGTTGGCGGGTTGCTCG	GAGCGGTACCCTCGAGTTAGCTTTGCTGAGAATG	*pVYNE(R)*
VC-OFP1	GCCTACTAGTGGATCCATGGCGATGGATCATCGT	GAGCGGTACCCTCGAGCTAGCCGCCATGGAGGAC	*pVYCE(R)*
GSK2-NLuc	TCGGTACCCGGGATCCATGGACCAGCCGGCGCCG	ACGAGATCTGGTCGACGCTCCCAGTATTGAAGAA	*pCAMBIA1300-35S-Nluc*
CLuc-OFP1	ACGCGTCCCGGGGCGGTACCATGGCGATGGATCATCGT	TCCATTTGTTGGATCCCTAGCCGCCATGGAGGAC	*pCAMBIA1300-35S-CLuc*
CLuc-GSK2	ACGCGTCCCGGGGCGGTACCATGGACCAGCCGGCGCCG	TCCATTTGTTGGATCCTTAGCTCCCAGTATTGAA	*pCAMBIA1300-35S-CLuc*
OFP1p-GUS	TACGCCAAGCTTGGCTGCAGGATCTCTACGGTGAAGGAT	GAATTCCCGGGGATCCTGGCAGCTACCACCAGTA	*pCAMBIA2391Z*
OFP1-CRISPR/CAS9-U3m	GGCAGCGATGGATCATCGTGGTGG	AAACCCACCACGATGATCCATCGC	*pYLCRISPR/Cas9-MH*
OFP1-OE	CGGTACCCGGGGATCCGATCTCTACGGTGAAGGAT	GCAGGTCGACTCTAGAAAAGGTGCACACTGATCA	*pCAMBIA2300*
DLT-RFP	TTTGGAGAGGACAGGGTACCATGTTGGCGGGTTGCTCG	TCGGAGGAGGCCATGGTACCGCTTTGCTGAGAATGTGA	*pCAMBIA2300-35S-RFP*
OFP1-GFP	GGGTACCCGGGGATCCATGGCGATGGATCATCGT	TAGTGTCGACTCTAGAGCCGCCATGGAGGACGAC	*pCAMBIA2300-35S-GFP*
OFP1-FLAG	GAGGCGCGCCGTCGACATGGCGATGGATCATCGT	TGTAGTCCATGTCGACGCCGCCATGGAGGACGAC	*pCAMBIA1300-35S-FLAG*

Supplemental Table 2. Primers used for quantitative PCR analyses.

**Name**	**Forward (5'-3')**	**Reverse (5'-3')**
**For qRT-PCR:**		
UBQ	GAGCCTCTGTTCGTCAAGTA	ACTCGATGGTCCATTAAACC
OFP1	AGCAGCTGCGTTGACACGT	GACGACCATCTCCACCAT
GA2ox-3	TGGTGGCCAACAGCCTAAAG	TGGTGCAATCCTCTGTGCTAAC
GA20ox-2	CCAATTTTGGACCCTACCGC	GAGAGAAGCCCAACCCAACC
GA3ox-2	TCCTCCTTCTTCTCCAAGCTCAT	GAAACTCCTCCATCACGTCACA
D2	AGCTGCCTGGCACTAGGCTCTACAGATCAC	ATGTTGTCGGAGATGAGCTCGTCGGTGAGC
D11	TTGGGTCATGGCATGGCAAGAGCAAGGA	TTGTTGCTGGAGCCAGCATTCCTCCTCT
DWARF	ATGGTGTTGGTGGCGATTGGGGTGGTTG	ATGTTGTTCCGCCCCAGGATGTCCAGCA
**For ChIP-qPCR:**		
OFP1 P1	ACCCTTGTCATTCCCTCTT	CGGAGTAGGGGTAGGTATG
OFP1 P2	GCTTGATGGGAAGGTGACT	TTTTGGGACGGAGGGATT
OFP1 P3	ACGCGTACTCAATGGTTG	TGTGTACGCGTACGCGCACT
DTL (coding region)	CCTGGCGTTCGAGTTCCA	TGGCGAGGACGCAGTTCA
ACTIN1 (intron)	TGGCATCTCTCAGCACATTC	GGCAAGCAACATTGTAAGCA
